# Effect of the Floor Level on the Probability of a Neurologically Favorable Discharge after Cardiac Arrest according to the Event Location

**DOI:** 10.1155/2019/9761072

**Published:** 2019-10-16

**Authors:** Han Joo Choi, Hyung Jun Moon, Won Jung Jeong, Gi Woon Kim, Jae Hyug Woo, Kyoung Mi Lee, Hyuk Joong Choi, Yong Jin Park, Choung Ah Lee

**Affiliations:** ^1^Department of Emergency Medicine, Dankook University Hospital, Cheonan-si, Chungcheongnam-do, Republic of Korea; ^2^Department of Emergency Medicine, College of Medicine, Soonchunhyang University, Cheonan-si, Chungcheongnam-do, Republic of Korea; ^3^Department of Emergency Medicine, Catholic University of Korea, St. Vincent's Hospital, Suwon, Gyeonggo-do, Republic of Korea; ^4^Department of Emergency Medicine, College of Medicine, Soonchunhyang University, Bucheon-si, Gyeonggi-do, Republic of Korea; ^5^Department of Emergency Medicine, Gil Medical Center, Gachon University College of Medicine, Incheon, Republic of Korea; ^6^Department of Emergency Medicine, Myongji Hospital, Goyangsi, Gyeonggo-do, Republic of Korea; ^7^Department of Emergency Medicine, Hanyang University Guri Hospital, Guri-si, Gyeonggo-do, Republic of Korea; ^8^Department of Emergency Medicine, Chosun University Hospital, Gwangju, Republic of Korea; ^9^Department of Emergency Medicine, Hallym University, Dongtan Sacred Heart Hospital, Hwaseong-si, Gyeonggi-do, Republic of Korea

## Abstract

As the number of people living in high-rise buildings increases, so does the incidence of cardiac arrest in these locations. Changes in cardiac arrest location affect the recognition of patients and emergency medical service (EMS) activation and response. This study aimed to compare the EMS response times and probability of a neurologically favorable discharge among patients who suffered an out-of-hospital cardiac arrest (OHCA) event while on a high or low floor at home or in a public place. This retrospective analysis was based on Smart Advanced Life Support registry data from January 2016 to December 2017. We included patients older than 18 years who suffered an OHCA due to medical causes. A high floor was defined as ≥3^rd^ floor above ground. We compared the probability of a neurologically favorable discharge according to floor level and location (home vs. public place) of the OHCA event. Of the 6,335 included OHCA cases, 4,154 (65.6%) events occurred in homes. Rapid call-to-scene times were reported for high-floor events in both homes and public places. A longer call-to-patient time was observed for home events. The probability of a neurologically favorable discharge after a high-floor OHCA was significantly lower than that after a low-floor OHCA if the event occurred in a public place (adjusted odds ratio (aOR), 0.58; 95% confidence intervals (CI), 0.37–0.89) but was higher if the event occurred at home (aOR, 1.40; 95% CI, 0.96–2.03). Both the EMS response times to OHCA events in high-rise buildings and the probability of a neurologically favorable discharge differed between homes and public places. The results suggest that the prognosis of an OHCA patient is more likely to be affected by the building structure and use rather than the floor height.

## 1. Introduction

Out-of-hospital cardiac arrest (OHCA) is a serious public health issue associated with poor outcomes [1]. Global incidence of this condition is high, and many countries have implemented programs and actions intended to improve the outcomes of affected patients [1, 2]. Previous studies have identified an association between survival after OHCA and emergency medical service (EMS) access time to patients, which is affected by the EMS system, ambulance density, and arrest location [3–5].

In 2018, 55% of the global population was estimated to reside in urban areas, and current projections suggest that this proportion will increase to 68% by 2050 [6]. Increases in population concentrations in urban areas have led to increases in the numbers of people who live in high-rise buildings. This demographic shift has exacerbated the issue of vertical patient access, as demonstrated by several studies reporting the negative outcomes of patients who experience OHCA in high-rise buildings. For example, significantly longer patient access times have been observed following ambulance calls for events that occurred ≥3 floors above ground, and an event site on a higher floor was associated with a lower 1-month neurologically favorable survival outcome after OHCA, compared to an event site on a lower floor [7, 8].

Despite the above findings, we did not assume that an increased vertical distance would always lead to a delayed EMS response time, as high-rise buildings tend to be densely populated and located in traffic centers. However, response times and patient outcomes might differ according to the structure of a high-rise building; a primarily residential building would have more independent spaces, compared to a public building. Therefore, this study aimed to compare the EMS response times and probability of a neurologically favorable discharge among patients who experienced an OHCA on a high floor (≥3 floors) in a home or in a public place.

## 2. Methods

### 2.1. Study Design and Setting

This retrospective cohort study was based on data included in the Smart Advanced Life Support (SALS) registry between January 2016 and December 2017. This is a prospective, population-based registry of OHCA cases that occurred in 18 urban and suburban areas in Korea, encompassing a total area of 7129.49 km^2^ and total population of 11.6 million inhabitants.

### 2.2. Data Source

SALS is a method of advanced field resuscitation administered by paramedics under direct, video communication-based medical direction [9]. However, the SALS registry includes all data of cardiac arrest cases, regardless of whether direct medical direction was provided. The SALS data set includes patient variables, resuscitation status, and outcome variables according to the international Utstein style for cardiac arrest (CA). The patient variables included age, sex, and comorbidities. The resuscitation variables included the initial electrocardiographic rhythm, witnessed CA, bystander cardiopulmonary resuscitation (CPR), and response time. The outcome variables included a return of spontaneous circulation (ROSC), survival admission, and discharge with a Cerebral Performance Category (CPC) score [1, 2].

### 2.3. Study Population

Patients older than 18 years who suffered an OHCA due to medical causes were included in this study. Patients who were not resuscitated because of obvious signs of death, a refusal of CPR, a do-not-resuscitate (DNR) status, or medically directed cessation of CPR; those whose CA was witnessed by 911-initiated first responders; and those who had no data records for height of CA were excluded. Although bystander or emergency medical technicians started chest compressions if there were no obvious signs of death, resuscitation was not performed if it was stopped immediately by medical directors because this indicated no possibility of resuscitation.

### 2.4. Main Outcomes

The primary outcome was hospital discharge with a neurologically favorable outcome after an OHCA on a high (≥3^rd^) floor in a home or public place. A neurologically favorable discharge was defined as a hospital discharge with a CPC score of 1 or 2 [10]. Additionally, we calculated the call-to-scene and call-to-patient times after OHCA for patients classified in the high-floor and low-floor (<3^rd^) groups according to the CA event location. We further analyzed the factors related to a neurologically favorable discharge among these patients according to event location.

### 2.5. Measurements

A home was defined as an apartment, condominium, house, or townhouse. All other locations were considered public places. A high floor was defined as the 3^rd^ or higher floor above ground, while a low floor was defined as the 2^nd^ or lower floor above ground or below ground. The call-to-scene time was calculated as the time interval from the initial call to 911 to the vehicle stop. The call-to-patient time was calculated as the interval from the initial call to 911 to the time when the defibrillator was activated.

### 2.6. Statistical Analysis

Statistical analyses were performed using SPSS, version 24.0 (IBM Corp., Armonk, NY, USA). The patient and resuscitation characteristics and outcomes were compared between the high- and low-floor groups. Chi-square and Mann–Whitney *U* tests were used to compare categorical and continuous variables, respectively. A multivariate logistic regression analysis adjusted for sex, age, bystander CPR, witnessed arrest, and shockable initial rhythm was used to estimate the adjusted odds ratios (aORs) and 95% confidence intervals (CIs) of clinical outcomes. No multicollinearity was detected, and all relevant interactions were considered. Additionally, the analysis of factors influencing discharge with a neurologically favorable outcome included targeted temperature management (TTM) as a confounder. A *P* value of <0.05 was considered statistically significant.

### 2.7. Ethical Statement

This study was approved by the institutional review board at Hallym University (approval number: HDT 2019-06-004).

## 3. Results

**Figure 1:**
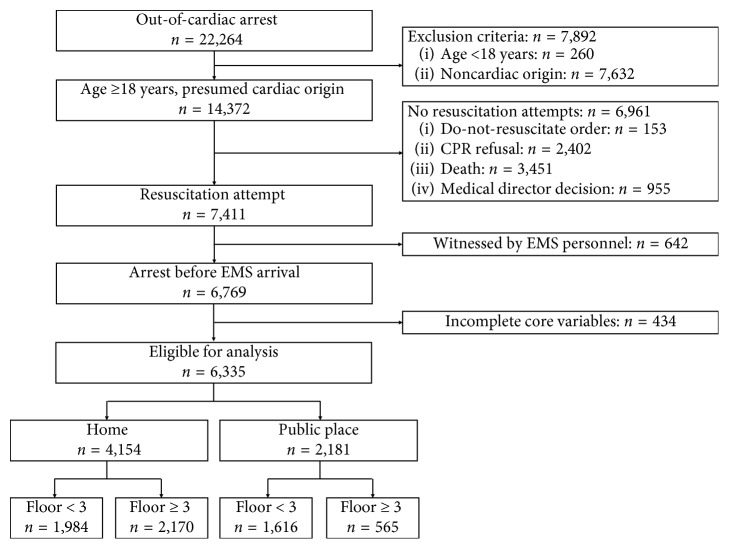
Overview of out-of-hospital cardiac arrest in home and public settings. CPR, cardiopulmonary resuscitation; EMS, emergency medical service.

The SALS database included 22,264 OHCA cases that occurred from January 2016 to December 2017. Of the 6,335 cases deemed eligible for the study, 4,154 (65.6%) occurred in a home. Furthermore, 52.2% of CA events in homes and 25.9% of events in public places occurred on a high floor (Figure 1). We found that OHCA events on a high floor in a home were more likely to involve a younger patient with a witness, shockable initial rhythm, and bystander CPR. OHCA events on a high floor in a public place were associated with an older age, nonshockable initial rhythm, and more frequent bystander CPR. The call-to-scene time was a median of 7 min, which is shorter on a high floor for events in both homes and public places. However, the call-to-patient time for home events was significantly longer on a high floor (a median of 9 min). Moreover, patients who experienced an OHCA event on a high floor at home were significantly more likely to experience a prehospital ROSC (25.39% vs. 21.22%), survival at admission (15.77% vs. 12.72%) and discharge (8.03% vs. 5.49%), and have favorable neurological outcome at discharge and have favorable neurological outcome at discharge (4.80% vs. 2.47%) after OHCA, compared to those who experienced OHCA on a low floor. In contrast, patients who experienced an OHCA event on a low floor in a public place were more likely to be alive on admission (22.94% vs. 16.31%), survive to discharge (15.27% vs. 8.88%), and have favorable neurological outcome at discharge (10.48% vs. 5.15%, Table 1).

Table 2 presents the aORs of clinical outcomes according to the arrest location after adjusting for sex, age, witness status, and bystander CPR. The prehospital ROSC, survival rate on admission, and survival rate at discharge after a high-floor OHCA event at home were positive, but not significant. In contrast, patients who experienced a high-floor OHCA in a public place had negative outcomes, with a significantly lower probability of survival discharge (aOR, 0.66; 95% CI, 0.47–0.92). In the multivariate analysis (Table 3), the likelihood of having favorable neurological outcomes at discharge after OHCA in a public place was significantly lower in the high-floor group (aOR, 0.58; 95% CI, 0.37–0.89), whereas at home, the probability of having favorable neurological outcomes at discharge was higher in the high-floor group (aOR, 1.40; 95% CI, 0.96–2.03). Male, younger age, witnessed arrest, bystander CPR, and TTM were associated with having favorable neurological outcomes at discharge regardless of CA location.

## 4. Discussion

Our results demonstrated that in a home setting, the floor height at which an OHCA event occurred did not affect the probability of a neurologically favorable outcome. In contrast, events that occurred on the lower floors of public places had a better prognosis than those that occurred on higher floors. Therefore, we cannot assume that the floor level in a high-rise building would necessarily impair the prognosis of an OHCA patient.

Early recognition and treatment access are key factors affecting patient survival after OHCA [5, 11]. Bystander CPR is considered the first step in both early recognition and access. Subsequently, a rapid EMS response time and resuscitation are considered significant predictors of patient survival after OHCA [12, 13]. Survival at discharge is also influenced by the allocation of EMS resources (e.g., ambulance density) [14]. The increasing urbanization of society and consequent increases in the numbers of users of high-rise buildings [15, 16] have led to concerns about delayed EMS responses due to issues of vertical access [8, 17, 18]. In South Korea, for example, apartment complexes comprising tower blocks have become increasingly common. In Seoul, approximately 80% of the population resides in apartment complexes, and these buildings account for 98% of all recent residential construction. Accordingly, the present study reported that 52.2% of high-floor OHCA events occurred in a home setting, a much higher proportion than those reported in other urban study areas (Osaka, 37.4%; Toronto, 22.4%) [8, 19]. However, it is doubtful whether an increase in vertical access time would increase the overall EMS response time or affect a patient's prognosis.

Patients who experience CA events on high floors are more likely than those on lower floors to be accessed and transported via elevators, which may be more advantageous than stairs. Additionally, skyscrapers tend to be located in city centers and are easily identifiable even if the building address has not been clearly stated. Moreover, in such cases, the ambulance will likely be required to travel a short distance to reach the building. Consistent with these factors, Conway et al. reported short call-to-scene times for incidents occurring in tall and large-volume buildings [20]. In this study, the call-to-scene times were shorter for events on high-levels in both public place and home settings. However, we found that the call-to-patient time varied in terms of the location of CA, with longer durations observed for low floors in home settings and high floors in public places. Consistent with a previous study, we found that a lack of access to an elevator that is sufficiently large to accommodate a stretcher, a lack of directional signage, and an entry code requirement are likely barriers to patient access in high-floor OHCA events at home [7]. Unlike previous studies, however, we observed shorter call-to-patient times in public places [7, 17, 19], which may be attributed to more accurate address reporting, open access ramps, and large elevators.

The demographic factors affecting a neurologically favorable discharge after OHCA varied greatly, depending on the floor height where the event occurred. OHCA events on higher floors at home tended to involve younger patients and were more frequently witnessed. Accordingly, the crude OR of a neurologically favorable discharge following OHCA at home was 1.94 times greater on a high floor than on a low floor. This finding was in contrast to previous studies [8, 18]. In Korea, a higher income level is associated with living in a high-rise apartment [21]. We therefore presume that residents of a high-rising building have a high level of economic health.

We observed more striking differences in the characteristics of patients who experienced OHCA in a public place according to the floor where the event occurred. Events on low floors were significantly more likely to involve younger men with an initial shockable rhythm, and therefore the crude OR of a neurologically favorable discharge was high (2.03). This result is attributed to the locations of adult daycare centers in Korea. These centers are frequently located on the upper floors of commercial buildings in urban areas, as they are convenient for caregivers and in proximity to private hospitals or clinics. Accordingly, patients who experienced OHCA events on higher floors of public places were older and had a bystander CPR rate 76.3% higher than the overall rate. Moreover, although the access times did not differ between the high and low floors, the high-floor group had aORs for a neurologically favorably discharge as low as 0.58. This result suggests that our analysis did not correct for the patients' health statuses and comorbidities prior to the CA, as the users of adult daycare centers would be more likely to have existing diseases and disabilities.

This study had several limitations in addition to the above-mentioned failure to control for the patients' pre-CA health statuses. Most notably, this was a retrospective study of registry data, and therefore we could not determine the reasons for delayed patient access or the structural features of the locations of CA events. Additionally, other confounding factors such as the quality of bystander CPR and automated external defibrillator use by bystanders may have influenced the association between the floor of patient contact and a neurologically favorable outcome after OHCA. However, we attempted to overcome these data limitations by combining EMS records with validated records of hospital treatments and outcomes.

## 5. Conclusion

We conclude that the nature of the setting (home vs. public place) affects the EMS response times to OHCA events in high-rise buildings, as well as the probability that a patient will achieve a neurologically favorable discharge following an event on a high floor. In other words, the patient's prognosis is more likely to be affected by the structure and use of the building, rather than by the floor height where the CA event occurred.

## Figures and Tables

**Table 1:** Demographic characteristics of out-of-hospital cardiac arrest patients according to the floor and location event.

	Home			Public place		
	<3^rd^ floor	≥3^rd^ floor	*P* value	<3^rd^ floor	≥3^rd^ floor	*P* value
Sex, male	1,214 (61.19)	1,357 (62.53)	0.388	1,208 (74.75)	314 (55.58)	<0.001
Age (years)	73.0 (60.0–80.0)	71.0 (56.0–80.0)	0.001	67.0 (54.0–79.0)	77.0 (61.8–84.0)	<0.001
Initial rhythm, shockable	245 (12.37)	322 (14.86)	0.021	522 (32.46)	93 (16.55)	<0.001
Witnessed	906 (46.11)	1,076 (50.00)	0.014	887 (55.86)	320 (57.04)	0.656
Bystander CPR	1,221 (61.82)	1,413 (65.39)	0.018	1,113 (69.48)	428 (76.29)	0.002
Call-to-scene time	8.0 (6.0–10.0)	7.0 (5.0–9.0)	<0.001	8.0 (6.0–11.0)	7.0 (5.0–8.0)	<0.001
Call-to-patient time	9.0 (7.0–12.0)	9.0 (8.0–12.0)	0.026	9.0 (7.0–12.0)	9.0 (7.0–12.0)	0.698
Outcomes						
Prehospital ROSC	421 (21.22)	551 (25.39)	0.002	518 (32.05)	158 (27.96)	0.073
Survival admission	252 (12.72)	341 (15.77)	0.005	370 (22.94)	92 (16.31)	0.001
Survival discharge	109 (5.49)	174 (8.03)	0.001	246 (15.27)	50 (8.88)	<0.001
Neurological favorable discharge	49 (2.47)	104 (4.80)	<0.001	169 (10.48)	29 (5.15)	<0.001

CPR, cardiopulmonary resuscitation; ROSC, return of spontaneous circulation.

**Table 2:** Adjusted odds ratios of clinical outcomes after a high-floor out-of-hospital cardiac arrest according to the event location.

Outcomes	Home	Public place
Prehospital ROSC	1.16 (0.99–1.35)	0.97 (0.77–1.21)
Survival admission	1.14 (0.94–1.37)	0.78 (0.59–1.01)
Survival discharge	1.24 (0.95–1.61)	0.66 (0.47–0.92)
Neurological favorable discharge	1.49 (1.04–2.15)	0.58 (0.38–0.90)

^*∗*^Adjusted for sex, age, presence of witnesses, and bystander cardiopulmonary resuscitation.

**Table 3:** Associations of various factors with a neurologically favorable discharge according to the cardiac arrest location.

Outcome	Home	Public place
Sex, male	2.11 (1.36–3.28)	2.15 (1.36–3.40)
Age (year)	0.94 (0.93–0.95)	0.95 (0.94–0.96)
Witnessed	4.70 (3.04–7.25)	5.26 (3.44–8.05)
Bystander CPR	2.08 (1.33–3.24)	2.08 (1.33–3.24)
TTM	7.23 (4.59–11.39)	4.28 (2.48–7.41)
≥3^rd^ floor	1.40 (0.96–2.03)	0.58 (0.37–0.89)

CPR, cardiopulmonary resuscitation; TTM, targeted temperature management.

## Data Availability

The SPSS data used to support the findings of this study are available from the corresponding author upon request.
